# PaCO_2_ is nonlinearly associated with NIV failure in patients with hypoxemic respiratory failure

**DOI:** 10.1186/s12890-024-03023-8

**Published:** 2024-05-10

**Authors:** Xiaoping Xu, Mengyi Ma, Yiwei Min, Wenhui Hu, Linfu Bai, Jun Duan

**Affiliations:** Department of Respiratory and Critical Care Medicine, The First Affiliated Hospital of Chongqing Medical University, Youyi Road 1, Yuzhong District, Chongqing, 400016 P. R. China

**Keywords:** Noninvasive ventilation, PaCO_2_, Restricted cubic splines, Hypoxemic respiratory failure

## Abstract

**Objective:**

To explore the association between PaCO_2_ and noninvasive ventilation (NIV) failure in patients with hypoxemic respiratory failure.

**Methods:**

A retrospective study was performed in a respiratory ICU of a teaching hospital. Patients admitted to ICU between 2011 and 2019 were screened. We enrolled the patients with hypoxemic respiratory failure. However, patients who used NIV due to acute-on-chronic respiratory failure or heart failure were excluded. Data before the use of NIV were collected. Requirement of intubation was defined as NIV failure.

**Results:**

A total of 1029 patients were enrolled in final analysis. The rate of NIV failure was 45% (461/1029). A nonlinear relationship between PaCO_2_ and NIV failure was found by restricted cubic splines (*p* = 0.03). The inflection point was 32 mmHg. The rate of NIV failure was 42% (224/535) in patients with PaCO_2_ >32 mmHg. However, it increased to 48% (237/494) in those with PaCO_2_ ≤ 32 mmHg. The crude and adjusted hazard ratio (HR) for NIV failure was 1.36 (95%CI:1.13–1.64) and 1.23(1.01–1.49), respectively, if the patients with PaCO_2_ >32 mmHg were set as reference. In patients with PaCO_2_ ≤ 32 mmHg, one unit increment of PaCO_2_ was associated with 5% reduction of NIV failure. However, it did not associate with NIV failure in patients with PaCO_2_ >32 mmHg.

**Conclusions:**

PaCO_2_ and NIV failure was nonlinear relationship. The inflection point was 32 mmHg. Below the inflection point, lower PaCO_2_ was associated with higher NIV failure. However, it did not associate with NIV failure above this point.

## Introduction

Noninvasive ventilation (NIV) is one of the main oxygen strategies for patients with acute hypoxemic respiratory failure. It can reduce respiratory rate, diminish the swing of esophageal pressure, and improve the oxygenation [1]. However, the rate of NIV failure is high in patients with hypoxemic respiratory failure. It ranges from 40 to 57% [2–4]. And patients with NIV failure are more likely to die in intensive care units (ICUs) [4]. Identification of the patients who are suitable for NIV is important.

In patients with de novo acute respiratory failure who required intubation for invasive mechanical ventilation, the time from NIV initiation to intubation was 78 ± 65 h in non-survivors versus 32 ± 24 h in survivors [5]. It indicates that delayed intubation may be associated with increased mortality. Another study also reported that pre-intubation NIV duration was associated with increased 30-day mortality [6]. Therefore, early identification of the patients who required invasive mechanical ventilation and early application of intubation is a promising strategy to reduce mortality. However, how to identify the patients at high risk for NIV failure is challenging. In current study, we aimed to explore the association between PaCO_2_ and NIV failure in patients with acute hypoxemic respiratory failure and determine the cutoff value to predict NIV failure.

## Methods

This was a retrospective study performed in a respiratory ICU of a teaching hospital. The study protocol was approved by the ethics committee of the First Affiliated Hospital of Chongqing Medical University (No. K2024-061-01). As the retrospective design, the informed consent was waived. Patients who admitted to our ICU between 2011 and 2019 were screened. The inclusion criteria were hypoxemia, use of NIV as a first-line therapy, and PaCO_2_ ≤ 45 mmHg before the use of NIV [7, 8]. The exclusion criteria were PaCO_2_ >45 mmHg before the use of NIV, heart failure as the primary reason for NIV, and presence of acute-on-chronic respiratory failure. And those patients with missing data were also excluded. In addition, some patients received high-flow nasal cannula (HFNC) after 2017. The use of NIV or HFNC was determined by the attending physicians based on their experience and the availability of the device.

Patients admitted to our ICU were managed following hospital protocols [9]. In our department, the dedicated noninvasive ventilator was used for all patients. The indications for NIV were as follows: (1) tachypnea (respiratory rate > 25 breaths/min), (2) clinical presentation of respiratory distress at rest (such as active contraction of the accessory inspiratory muscles or paradoxical abdominal motion), or (3) PaO_2_ < 60 mmHg at room air or PaO_2_/FiO_2_ < 300 mmHg with supplemental oxygen. If supplemental oxygen was used, FiO_2_ was estimated as follows: FiO_2_ (%) = 21 + 4×fow (L/min) [10, 11]. The contraindications for NIV were as follows: (1) facial or nasal abnormalities, (2) recent gastric or esophageal surgery, (3) active upper gastrointestinal bleeding, (4) high risk for aspiration, (5) unable to clear sputum, (6) hemodynamic instability without response to fluids or vasoactive agents, and (7) lack of cooperation [12]. However, the use of NIV was at the physician’s discretion.

NIV was managed by attending physicians, respiratory therapists, and nurses. A face mask was the main interface for NIV treatment. A nasal mask was the secondary choice if the patient failed to tolerate the face mask. Selection of the mask was based on the patient’s facial or nasal type. The straps of the mask were kept as tight as possible while remaining comfortable to the patient. Inspiratory pressure was initially set at 8 or 10 cmH_2_O and then increased in increments of 2 cmH_2_O to achieve the best control of dyspnea. Expiratory pressure was initially set at 4 cmH_2_O and then increased to maintain the patency of the alveoli. It was gradually increased until the SpO_2_ or PaO_2_ reached a plateau. However, it also balanced patient’s tolerance. Usually the expiratory pressure was kept between 4 and 10 cmH_2_O. FiO_2_ was set to achieve peripheral oxygen saturation greater than 92%. If the respiratory conditions were gradually improved, the liberation from NIV was performed. However, intubation was performed if the respiratory conditions were progressively deteriorated.

We collected the age, gender, diagnosis, and disease severity from the medical records. Disease severity was assessed by APACHE II score. The heart rate, respiratory rate, systolic blood pressure, diastolic blood pressure, pH, PaCO_2_, and PaO_2_/FiO_2_ were also extracted from the medical records. NIV failure and hospital mortality were also recorded.

The Statistical Product and Service Solutions (version 25.0) and R (version 4.3.2) were used to analyze the data. Continuous variables were presented using the mean and standard deviation, whereas categorical variables were presented as percentages. A nonlinear relationship between PaCO_2_ and NIV failure was analyzed by restricted cubic splines. If a nonlinear relationship was found, a cutoff value was determined at the inflection point. Cox proportional hazards regression was used to explore the association between low PaCO_2_ and NIV failure. The hazard ratio (HR) for NIV failure was adjusted by confounders. Kaplan-Meier curves were also used to explore the cumulative incidence of NIV failure between two groups. A *p* value <0.05 suggests statistical significance.

## Results

**Fig. 1:**
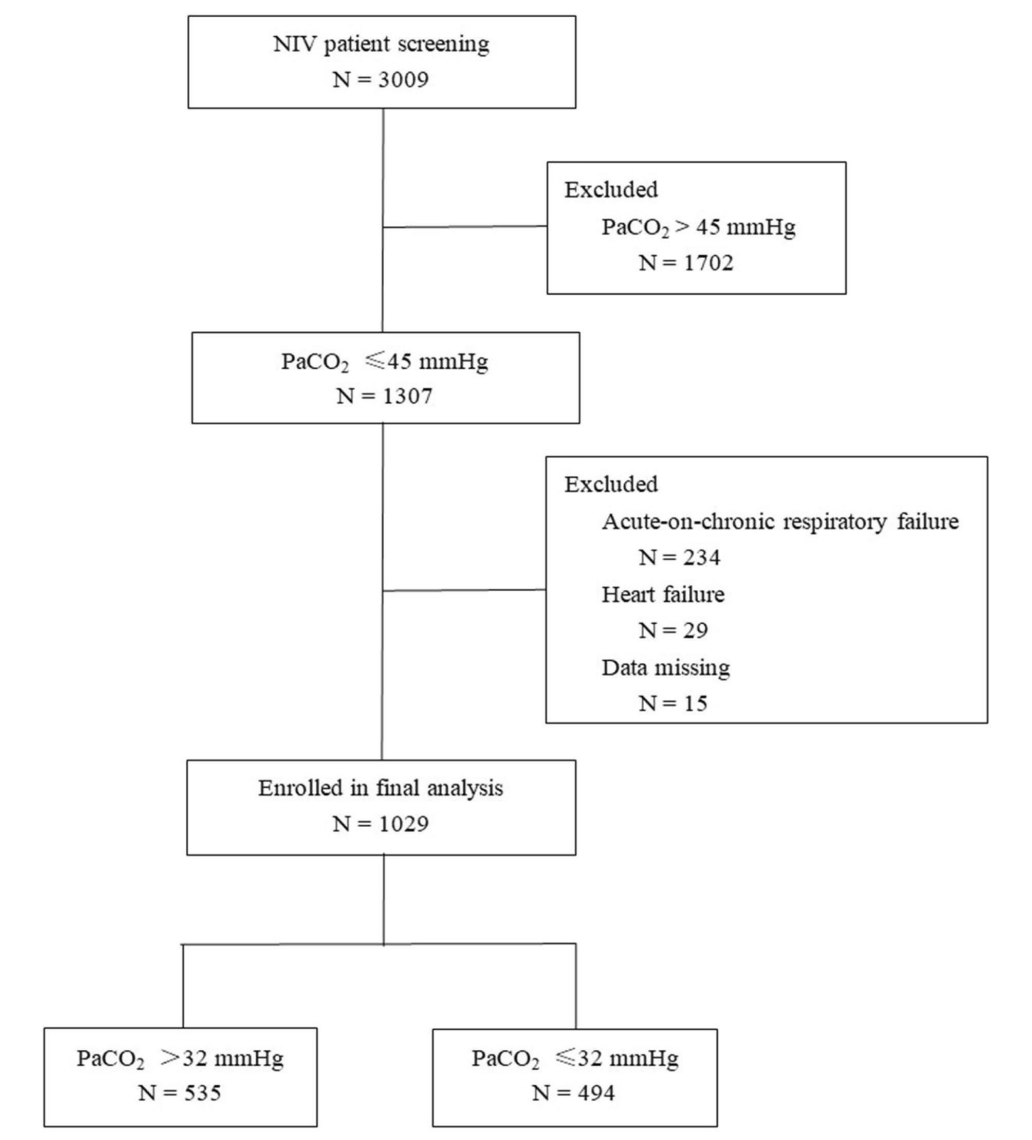
Patient screening

We screened 3009 patients in current study (Fig. 1). After exclusion of the ineligible patients, a total of 1029 patients were enrolled in final analysis. The mean age was 64 years (Table 1). The main diagnosis was pneumonia (accounting for 57%), and acute respiratory distress syndrome (ARDS) was the secondary diagnosis (accounting for 20%). Before the use of NIV, the mean respiratory rate was 33 breaths/min, mean PaCO_2_ was 33 mmHg, and mean PaO_2_/FiO_2_ was 156 mmHg. Four hundred and sixty-one patients (45%) experienced NIV failure and 338 patients (33%) died in hospital.

**Table 1:** Demographics

	Total cohort *N* = 1029	PaCO_2_ >32 mmHg *N* = 535	PaCO_2_ ≤32 mmHg *N* = 494	*p*
Age, years	64 ± 17	65 ± 18	64 ± 16	0.37
Male, %	724 (70%)	371 (69%)	353 (72%)	0.50
APACHE II score	16 ± 5	16 ± 5	17 ± 5	< 0.01
Diagnosis				
Pneumonia	586 (57%)	309 (58%)	277 (56%)	0.61
ARDS	210 (20%)	106 (20%)	104 (21%)	0.64
Pulmonary embolism	39 (4%)	15 (3%)	24 (5%)	0.10
Sepsis	22 (2%)	8 (2%)	14 (3%)	0.20
Other	172 (17%)	97 (18%)	75 (15%)	0.21
Data collected before NIV				
Heart rate, beats/min	116 ± 23	114 ± 23	118 ± 24	0.01
Respiratory rate, breaths/min	33 ± 8	32 ± 8	34 ± 7	< 0.01
Systolic blood pressure, mmHg	133 ± 27	135 ± 27	130 ± 26	0.01
Diastolic blood pressure, mmHg	78 ± 16	79 ± 17	78 ± 16	0.17
pH	7.44 ± 0.08	7.43 ± 0.08	7.46 ± 0.08	< 0.01
PaCO_2_, mmHg	33 ± 6	38 ± 4	27 ± 4	< 0.01
PaO_2_/FiO_2_, mmHg	156 ± 72	161 ± 79	151 ± 64	0.03
Data collected after 1–2 h of NIV				
Heart rate, beats/min	109 ± 23	107 ± 22	111 ± 23	< 0.01
Respiratory rate, breaths/min	30 ± 8	29 ± 8	31 ± 8	< 0.01
Tidal volume, mL	479 ± 176	455 ± 164	505 ± 184	< 0.01
Minute ventilation, L	14.9 ± 7.7	13.5 ± 7.1	16.4 ± 8.1	< 0.01
Systolic blood pressure, mmHg	127 ± 24	130 ± 24	124 ± 23	< 0.01
Diastolic blood pressure, mmHg	72 ± 14	73 ± 13	72 ± 14	0.75
pH	7.44 ± 0.08	7.44 ± 0.08	7.44 ± 0.09	0.33
PaCO_2_, mmHg	34 ± 9	38 ± 8	30 ± 7	< 0.01
PaO_2_/FiO_2_, mmHg	177 ± 85	184 ± 92	169 ± 77	< 0.01
Outcomes				
NIV failure, %	461 (45%)	224 (42%)	237 (48%)	0.05
Mortality, %	338 (33%)	170 (32%)	168 (34%)	0.47

ARDS = acute respiratory distress syndrome, NIV = noninvasive ventilation

**Fig. 2:**
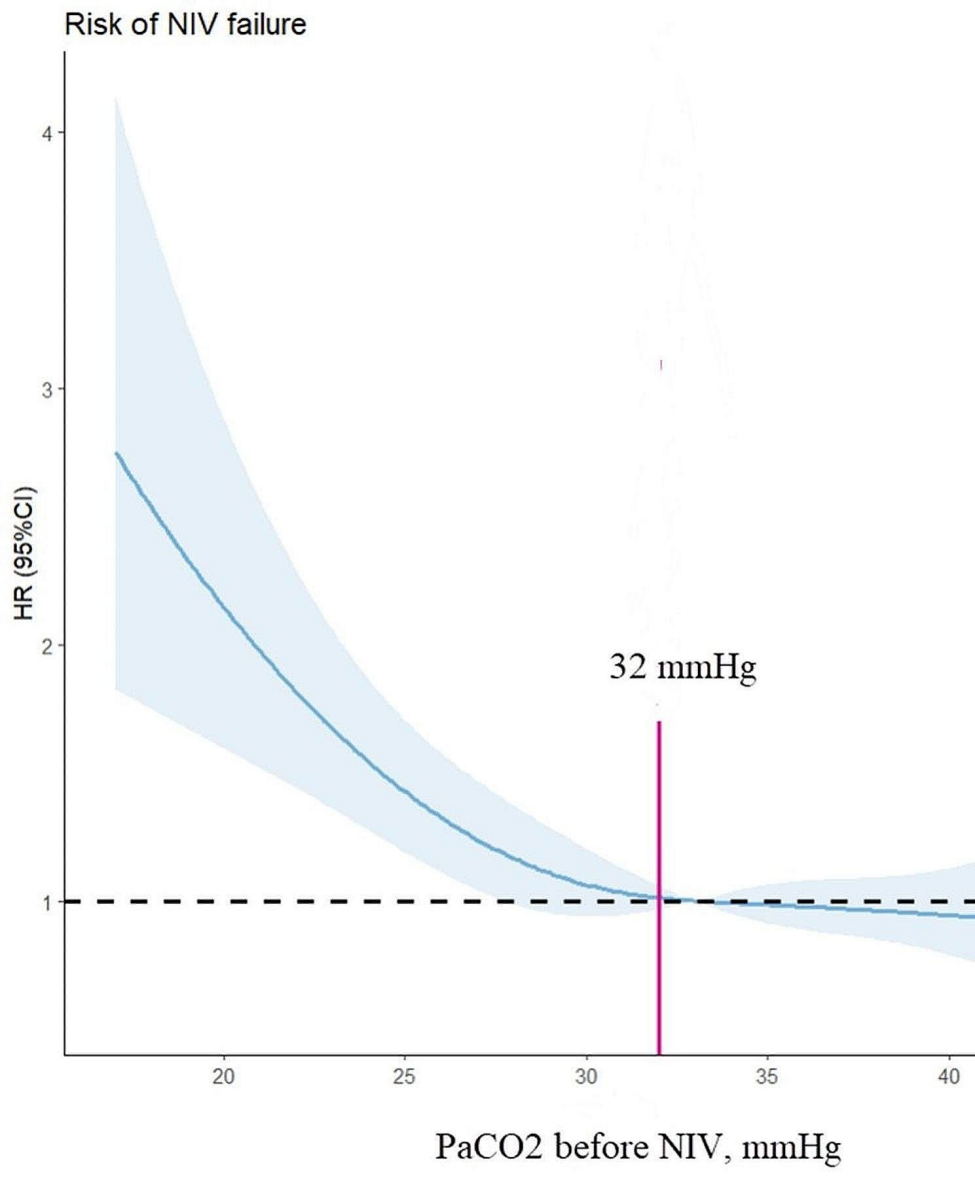
Relationship between PaCO_2_ and NIV failure

A nonlinear relationship between PaCO_2_ and NIV failure was identified by restricted cubic splines (*p* = 0.03). The inflection point was 32 mmHg (Fig. 2). Four hundred and ninety-four patients (48%) had PaCO_2_ ≤ 32 mmHg before the use of NIV (Table 1). Patients with PaCO_2_ ≤ 32 mmHg had higher APACHE II score, higher respiratory rate, higher heart rate, and lower oxygenation than those with PaCO_2_ >32 mmHg.

**Fig. 3:**
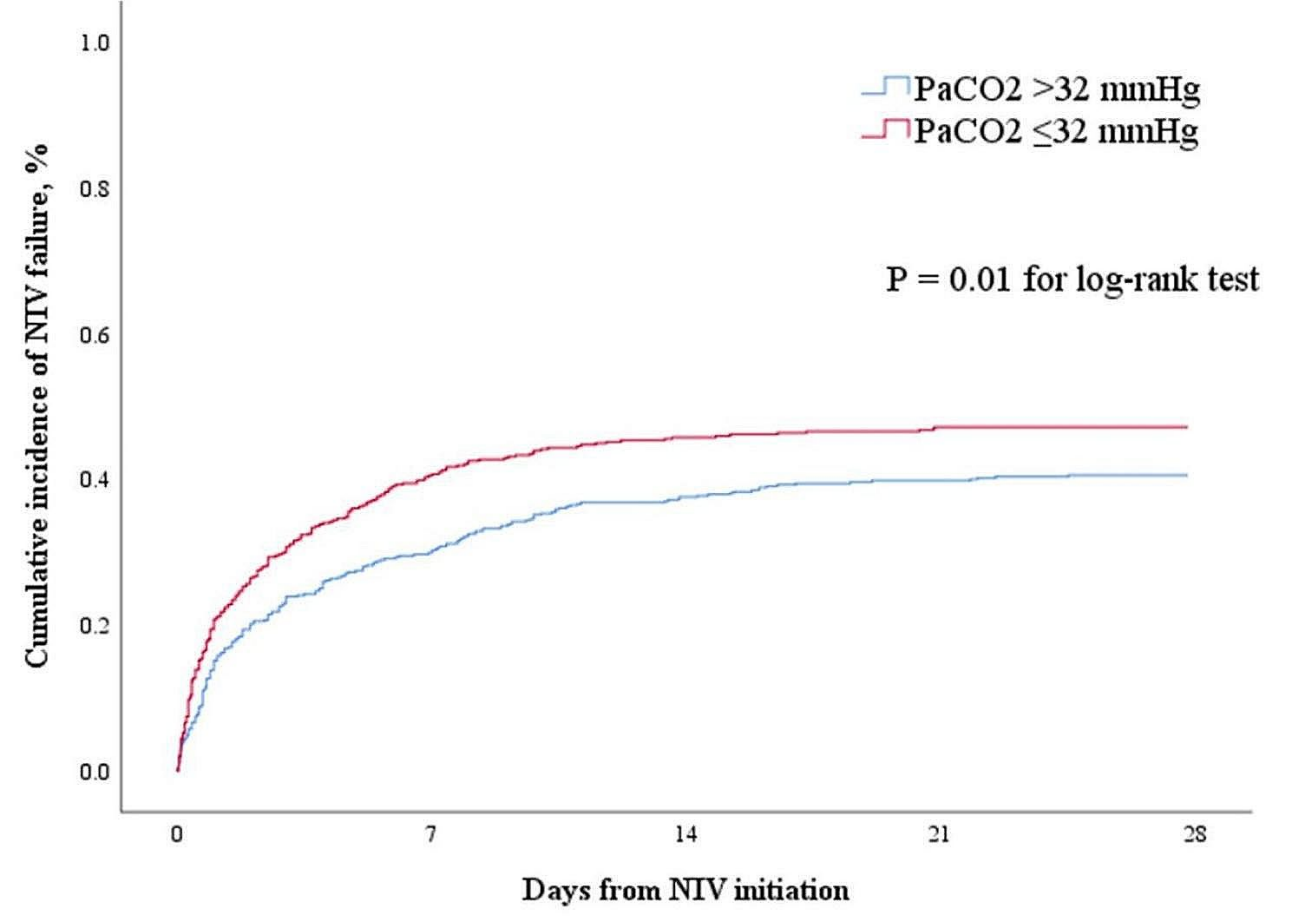
The cumulative incidence of NIV failure in patients with PaCO_2_ more than and less than 32 mmHg

The rate of NIV failure was 48% in patients with PaCO_2_ ≤ 32 mmHg versus 42% in those with PaCO_2_ >32 mmHg (*p* = 0.05). The crude HR for NIV failure was 1.36 (95%CI: 1.13–1.64) if the patients with PaCO_2_ >32 mmHg were set as reference (Table 2). When it was adjusted by age, sex, diagnosis, APACHE II score, respiratory rate, heart rate, systolic blood pressure, diastolic blood pressure, pH and PaO_2_/FiO_2_, the HR was 1.23 (95%CI: 1.01–1.49). The cumulative incidence of NIV failure within 28 days was also much higher in patients with PaCO_2_ ≤ 32 mmHg (*p* = 0.01 for log-rank test, Fig. 3). After 1–2 h of NIV, the crude and adjusted HR for NIV failure was 1.28 (95%CI: 1.06–1.53) and 1.32 (1.08–1.60), respectively, if the PaCO_2_ was still less than 32 mmHg.

**Table 2:** HR (95% CIs) for NIV failure

	Crude HR (95%CI)	*p*	Adjusted HR (95%CI)	*p*
PaCO_2_>32 mmHg	Reference		Reference	
PaCO_2_ ≤ 32 mmHg	1.36 (1.13–1.64)	< 0.01	1.23 (1.01–1.49) #	0.04

HR = hazard ratio, CI = confidence interval, NIV = noninvasive ventilation

# It was adjusted by age, sex, diagnosis, APACHE II score, and respiratory rate, heart rate, systolic blood pressure, diastolic blood pressure, pH and PaO_2_/FiO_2_ before NIV.

In patients with PaCO_2_ >32 mmHg, the adjusted HR for NIV failure was 0.97 (95%CI: 0.94–1.01, *p* = 0.16) per one unit increment of PaCO_2_, indicating no association between PaCO_2_ and NIV failure (Table 3). However, in patient with PaCO_2_ ≤ 32 mmHg, the adjusted HR for NIV failure was 0.95 (95%CI: 0.92–0.98, *p* < 0.01) per one unit increment of PaCO_2_. The rate of NIV failure was 65.7%, 55.9%, 46.6%, 39.3%, and 42.7% in patients with PaCO_2_ less than 20 mmHg, 20.1–25 mmHg, 25.1–30 mmHg, 30.1–35 mmHg, and 35.1–45 mmHg, respectively (*p* < 0.01 between groups, Fig. 4).

**Table 3:** HR (95% CIs) for NIV failure in different subgroups

	Crude HR (95%CI)	*p*	Adjusted HR (95%CI)	*p*
PaCO_2_ per one unit increment in patients with PaCO_2_ >32 mmHg	0.99 (0.96–1.03)	0.81	0.97 (0.94–1.01)#	0.16
PaCO_2_ per one unit increment in patients with PaCO_2_ ≤ 32 mmHg	0.94 (0.91–0.97)	< 0.01	0.95 (0.92–0.98)#	< 0.01

HR = hazard ratio, CI = confidence interval, NIV = noninvasive ventilation

# It was adjusted by age, sex, diagnosis, APACHE II score, and respiratory rate, heart rate, systolic blood pressure, diastolic blood pressure, pH and PaO_2_/FiO_2_ before NIV.

**Fig. 4:**
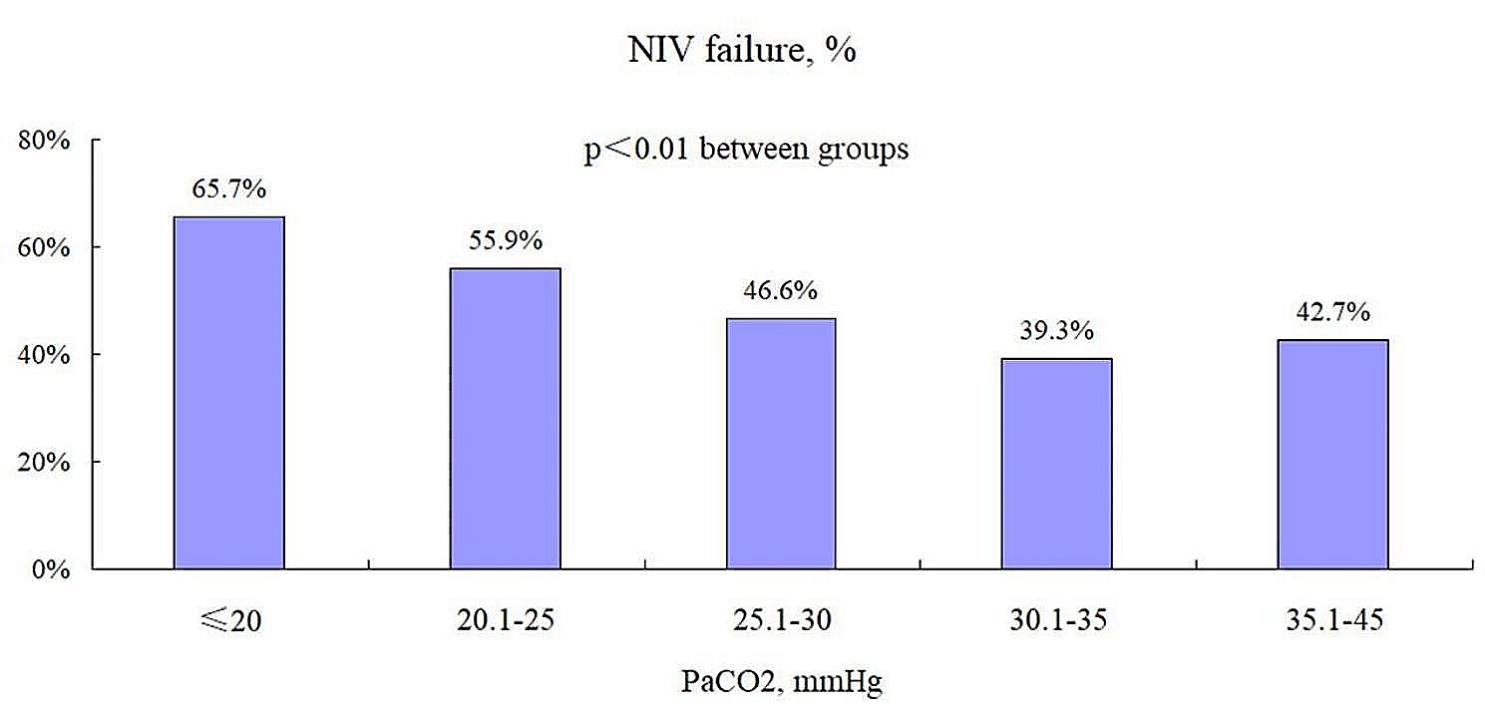
The rate of NIV failure in patients with different subgroups classified by PaCO_2_

## Discussion

This study showed that the relationship between PaCO_2_ and NIV failure was nonlinear in patients with hypoxemic respiratory failure. The inflection point was 32 mmHg. When the PaCO_2_ was less than 32 mmHg, lower PaCO_2_ was associated with higher NIV failure. However, there is no association between PaCO_2_ and NIV failure if the PaCO_2_ more than 32 mmHg in patients with hypoxemic respiratory failure.

To the best of our knowledge, this is the first study to explore the relationship between PaCO_2_ and NIV failure in patients with hypoxemic respiratory failure. Previous studies have shown that many variables were associated with NIV failure such as respiratory rate, pH, PaO_2_/FiO_2_, disease severity, and so on [13–15]. Our study demonstrated that PaCO_2_ ≤ 32 mmHg was association with increased NIV failure. This variable can be served as another predictor to predict NIV failure in patients with hypoxemic respiratory failure. Use of NIV in patients with low PaCO_2_ should be cautious and frequent assessment of the efficacy of NIV is required to avoid delayed intubation.

PaCO_2_ reflects the ventilation status. Low PaCO_2_ is associated with excess ventilation. A previous study reported by Carteaux and colleagues shown that patients with high minute ventilation or high tidal volume were more likely to experience NIV failure [8]. In that study, the ICU ventilator with double circuits was used to deliver gas to the patient. And the expired tidal volume was recorded. However, we only used dedicated noninvasive ventilators with single circuit to deliver gas. The expired tidal volume is unavailable. These ventilators only calculated the estimated tidal volume. The accuracy of tidal volume was based on the algorithms of each ventilator. It was also influenced by air leak around the mask. Since the tidal volume estimated by dedicated noninvasive ventilator is not accurate, use of PaCO_2_ as a predictor to predict NIV failure is complementary to tidal volume.

PaCO_2_ can partly reflect the respiratory drive. High respiratory drive leads to strong muscle contraction and large negative pleural swings, which leads to lung injury [16–18]. As preservation of spontaneous breathing is required for NIV, it is named as self-inflicted lung injury [19]. Exposure to high respiratory drive is associated with increased severity of lung injury. This may be another reason for the association between low PaCO_2_ and high rate of NIV failure.

Our study has several limitations. Firstly, the use of NIV was at the physician’s discretion. This may delay the timing of intubation. Secondly, we only demonstrated that patients with PaCO_2_ less than 32 mmHg were associated with increased NIV failure. This did not mean that NIV should not be used in these patients. A randomized controlled trial is encouraged to determine this issue. Thirdly, use of HFNC may influence patient allocation. However, only a small number of HFNC devices were introduced to our department at the end stage of the study period. Use of HFNC was at physician’s discretion and the availability of the device. Therefore, the selection bias using NIV or HFNC is small.

## Conclusion

Nonlinear relationship between PaCO_2_ and NIV failure was found in patients with hypoxemic respiratory failure. When the PaCO_2_ was less than 32 mmHg, lower PaCO_2_ was associated with higher NIV failure. However, there was no association between PaCO_2_ and NIV failure if the PaCO_2_ was more than 32 mmHg in patients with hypoxemic respiratory failure.

## Data Availability

The dataset used and/or analyzed during the current study is available from the corresponding author on reasonable request.

## Abbreviations

ICU: intensive care unit
ARDS: acute respiratory distress syndrome
NIV: noninvasive ventilation
HR: hazard ratio
CI: confidence interval
HFNC: high-flow nasal cannula
